# Frequently asked questions regarding SARS-CoV-2 in cancer patients—recommendations for clinicians caring for patients with malignant diseases

**DOI:** 10.1038/s41375-020-0832-y

**Published:** 2020-05-01

**Authors:** Marie von Lilienfeld-Toal, Jörg Janne Vehreschild, Oliver Cornely, Livio Pagano, Francesca Compagno, EHA Infectious Disease Scientific Working Group, Antonio Pagliuca, Hamdi Akan, Katrin Lagrou, Livio Pagano, Martin Hoenigl, Nikolai Klimko, Oliver Cornely, Paul Verweij, Rafael Duarte, Stefan Zimmerli, Stephane Bretagne, Zdenek Racil, Hans H. Hirsch

**Affiliations:** 1 Klinik für Innere Medizin II, Abteilung für Hämatologie und Internistische Onkologie, Universitätsklinikum Jena, Jena, Germany; 2 Leibniz-Institut für Infektionsbiologie und Naturstoff Forschung, Hans-Knöll Institut, Jena, Germany; 3 Department of Internal Medicine, Hematology/Oncology, Goethe University Frankfurt, Frankfurt am Main, Germany; 4 Department I of Internal Medicine, Excellence Center for Medical Mycology (ECMM), Faculty of Medicine and University Hospital Cologne, University of Cologne, Cologne, Germany; 5 German Centre for Infection Research (DZIF), Partner Site Bonn-Cologne, Cologne, Germany; 6 Cologne Excellence Cluster on Cellular Stress Responses in Aging-Associated Diseases (CECAD), University of Cologne, Cologne, Germany; 7 Clinical Trials Centre Cologne (ZKS Köln), University of Cologne, Cologne, Germany; 8 EHA Infectious Diseases Scientific Working Group, Cologne, Germany; 9 Department of Hematology, Fondazione Policlinico Universitario A. Gemelli—IRCCS, Universita Cattolica del Sacro Cuore, Rome, Italy; 10 Pediatric Hematology Oncology, Fondazione IRCCS Policlinico San Matteo, Pavia, Italy; 11 Clinical Virology, Laboratory Medicine, University Hospital Basel, Basel, Switzerland; 12 Transplantation & Clinical Virology, Department Biomedicine, University of Basel, Basel, Switzerland; 13 Infectious Diseases & Hospital Epidemiology, University Hospital Basel, Basel, Switzerland; 14 Department of Haematological Medicine, King’s College Hospital NHS Foundation Trust, London, UK; 15 Hematology Clinical Research Unit, Ankara University, Cebeci Campus, Ankara, Turkey; 16 Laboratory of Clinical Bacteriology and Mycology, Department of Microbiology, Immunology and Transplantation, Excellence Center for Medical Mycology (ECMM), KU Leuven, Leuven, Belgium; 17 Department of Internal Medicine, Section of Infectious Diseases and Tropical Medicine, Medical University of Graz, Graz, Austria; 18 Department of Clinical Mycology, Allergology and Immunology, North Western State Medical University, St. Petersburg, Russia; 19 Department of Medical Microbiology, Excellence Center for Medical Mycology (ECMM), Center of Expertise in Mycology Radboudumc/CWZ, Nijmegen, The Netherlands; 20 Department of Hematology, Hospital Universitario Puerta de Hierro, Madrid, Spain; 21 Department for Infectious Diseases, University Hospital Bern, Bern, Switzerland; 22 Institut Pasteur, Molecular Mycology Unit, Department of Mycology, CNRS UMR2000, Laboratoire de Parasitologie-Mycologie, Hôpital Saint-Louis, Groupe Hospitalier Lariboisière, Saint-Louis, Fernand Widal, Assistance Publique-Hôpitaux de Paris (AP-HP), Université Sorbonne Paris Cité, Paris, France; 23 Institute of Hematology and Blood Transfusion, Prague, Czech Republic

**Keywords:** Diseases, Health care

## Abstract

Since early 2020, the SARS-CoV-2 pandemic has a massive impact on health care systems worldwide. Patients with malignant diseases are assumed to be at increased risk for a worse outcome of SARS-CoV-2 infection, and therefore, guidance regarding prevention and management of the infection as well as safe administration of cancer-therapy is required. Here, we provide recommendations for the management of patients with malignant disease in the times of COVID-19. These recommendations were prepared by an international panel of experts and then consented by the EHA Scientific Working Group on Infection in Hematology. The primary aim is to enable clinicians to provide optimal cancer care as safely as possible, since the most important protection for patients with malignant disease is the best-possible control of the underlying disease.

## Introduction: SARS-CoV-2

SARS-CoV-2 is a novel betacoronavirus that has been first identified in China in winter 2019 as the cause of a severe inflammatory coronavirus infectious disease (COVID-19) of the respiratory tract. Because its genome is closest related to previously identified CoVs in bats, SARS-CoV-2 was originally thought to represent a zoonosis. However, its rapid spread in the human population suggests that SARS-CoV-2 is surprisingly well adapted to the human host hence behaving like a new member of the community-acquired respiratory viruses (CARVs). Similar to other CARVs, the clinical presentation can range from a- and oligosymptomatic to full-blown influenza-like illness and primary viral pneumonia. For the purpose of these clinical recommendations, manifestations of upper respiratory tract infectious disease are diagnosed if there is at least one of the following: cough, coryza, sore throat, shortness of breath AND a systemic symptom/sign such as fever/malaise/myalgia AND laboratory confirmation by nucleic acid testing (NAT). Conversely, lower respiratory tract infectious disease (LRTID) is diagnosed in patients with tracheitis and bronchitis, whereby dyspnea and/or declining oxygen saturation at ambient air often predict bi-lateral ground glass infiltrates seen earliest on CT-scans, hence identifying viral pneumonia. Notably, LRTID may occur early or late in the course and may rapidly progress to life-threatening respiratory failure because of massive impairment of gas-exchange. In addition, multi-organ failure affecting renal and cardiac function have been described. This life-threatening complication is thought to be due to a hyperinflammatory process compatible with hemophagocytic lymphohistiocytosis (HLH) [1, 2] in addition to hypercoagulopathy leading to pulmonary embolism [3].

In general, CARVs follow a seasonality with influenza, respiratory syncytial and parainfluenza viruses being the most prominent ones [4]. Influenza and RSV can be treated with specific antivirals, which is essential for the care of infected cancer patients. Importantly, cancer patients with CARV-infections are prone to co-infections, and bacterial/fungal superinfections are the primary cause of CARV-associated mortality in cancer patients [5].

Infection with SARS-CoV-2 has been described to occur after an incubation period of about 3–5 days, with the most common symptoms being raised temperature/fever and cough [6]. Of note, the peak of clinical disease is in the 2nd week with an increase in inflammation [7]. It is not clear if cancer is a general risk factor for infection [6, 8, 9], but old age, hypertension, and diabetes have clearly been defined as risk factors for worse outcome [7]. Cancer patients should also be assumed to have a worse prognosis [8] and preliminary experience from Italy suggests that mortality in patients with hematological malignancy may be as high as 20% (Livio Pagano, personal communication). However, with regard to the present or upcoming outbreak of SARS-CoV-2 accurate estimates regarding mortality and spread are almost impossible to give since most factors influencing the epidemiology are still unknown [10].

## Specific aspects for hematologists and oncologists

Clinicians caring for cancer patients during the SARS-CoV-2 pandemic face a variety of challenges including the management of cancer patients infected with SARS-CoV-2 as well as coping with a drastically changed work environment due to emergency measures, staff sicknesses and expected shortages of drugs, blood products, and equipment for personal protection amongst others. In this setting it appears wise to implement center-specific standard-operating-procedures to define altered responsibilities and access to care. Depending on the magnitude of changes within a certain region, prioritization with regard to anti-cancer therapy may be necessary to ensure the safe administration of potentially dangerous and toxic treatments. An example of these changed working conditions may be that centers may automatically admit all patients presenting with febrile temperature to an area dedicated to the treatment of COVID-19 patients. If, however, a cancer patient treated in the outpatient clinic presents with febrile neutropenia and is admitted to the COVID-19 dedicated area, the cancer patient may actually be at higher risk and not receive the adequate therapy within the required time frame in this emergency setting. It seems advisable to envisage the situation of delivering care in a COVID-19-dedicated area during the time of the most profound immunosuppression caused by anti-cancer treatment. Some elective therapies leading to profound immunosuppression later on may be re-evaluated under the consideration that the management of complications may be hampered because of emergency measures. On the other hand, it is vital to ensure appropriate cancer therapy as much as possible, especially in patients with curative options and rapid proliferation of their disease. Therefore, the individual situation of cancer patients needs to be considered when implementing emergency measures.

We have addressed some potential questions relevant for clinicians caring for cancer patients in the following. We do not address the topic of stem cell transplantation and cell therapy, since this is the focus of guidelines prepared by the European Society for Blood and Marrow Transplantation which can be accessed under https://www.ebmt.org/ebmt/news/coronavirus-disease-covid-19-ebmt-recommendations-update-march-23-2020. Also, we do not provide specific recommendations as to structural changes within departments caring for cancer patients because geographical differences are too large to make generalized statements. However, individual treatment decisions will have to take these aspects into account and clinicians may have to deviate from these recommendations due to reduced capacities or other structural impediments.

**(1) What is the risk for the patient to become infected with SARS-CoV-2 and what is the risk for the patient to have a severe disease course?**

Currently available data does not show a different rate of infection in patients with a malignant disease compared to healthy members of the population [6, 7]. This is mostly due to the fact that cancer patients as well as healthy individuals are immunologically naive to this novel pathogen. Regarding the course of the infection, underlying malignant disease has not been identified as an independent risk factor in multivariate analysis so far [7]. However, severely immunosuppressed patients generally have a higher risk of developing complications in CARV infections [11] and it should be assumed that cancer patients are at risk of a more severe course of COVID-19 as well [8]. In the absence of more specific data, potential risk factors for a severe course of the disease should be assumed as for other CARV-infections: severe immunodeficiency, lymphopenia, long and profound neutropenia, and older age [11]. As lymphopenia is a risk factor for LRTID and mortality in CARV infections in SCT-recipients and SARS-CoV-2 appears to cause or be associated with lymphopenia, patients with pre-existing severe lymphopenia (<0.2 × 10^9^/L) may in time be shown to have an increased risk for a severe course of COVID-19 as well [7, 11]. Clinicians should also be aware that cancer patients generally shed CARV longer than immunocompetent people [12] and this is probably true for this novel coronavirus as well.

**(2) What can be done to prevent COVID-19?**

The most important measures are general contact precautions including hand hygiene. Patients should be counselled regarding current recommendations of their local and national health authorities. It is strongly advised that patients with active malignant disease or currently on treatment should stay away from any social gatherings, especially in high-risk areas [8]. Also, all symptomatic family members or those at high risk of early infection with SARS-CoV-2 should stay away from patients with active malignant disease or currently on treatment regardless of the risk of corona. The aim is to prevent any infection with CARV. Face masks can be offered for situations when contact isolation is difficult, but patients should be aware of a possibly false sense of security and never rely only on them [13].

Good general health should be reinforced in cancer patients. Patients should be advised regarding the importance of good general health: stop smoking, reduce weight loss, physiotherapy, sufficient supply of vitamins like vitamin C and D, and the treatment of any iron-deficiency.

Patients with secondary immunodeficiencies should be evaluated if they require intravenous immunoglobulins (ivIg) as recommended by EMA (recurrent infections in patients with IgG < 4 g/l due to secondary immunodeficiency) [14]. Physicians need to be aware that ivIg are NOT specifically effective against SARS-CoV-2 because of lack of specific antibodies within the product, but they help to generally restore a defective immune response and help to prevent additional (for example bacterial) infections. As time elapses there is a high probability that new preparations from immune donors will include protective antibodies as well.

Prophylactic antibiotics and generous implementation of G-CSF seem a logical attempt to avoid bacterial superinfections and shorten the duration of severe immunosuppression. However, prior exposure to antibiotics has been associated with adverse outcome in CARV-infections in stem-cell transplant recipients [15]. Also, in geographical areas with a high rate of multi-drug resistant bacteria, antibiotic prophylaxis is unlikely to work. G-CSF on the other hand is known to be associated with the risk of hyperinflammation during regeneration. Thus, we strongly recommend not to implement additional antimicrobial prophylaxis on top of recent recommendations by current guidelines concerning the use of antibiotics and G-CSF [16–18]. In contrast, vaccination against influenza and especially pneumococci should be recommended for all cancer patients as per current guidelines [19, 20].

**(3) Who should have therapy for malignant disease deferred or interrupted?**

**Fig. 1:**
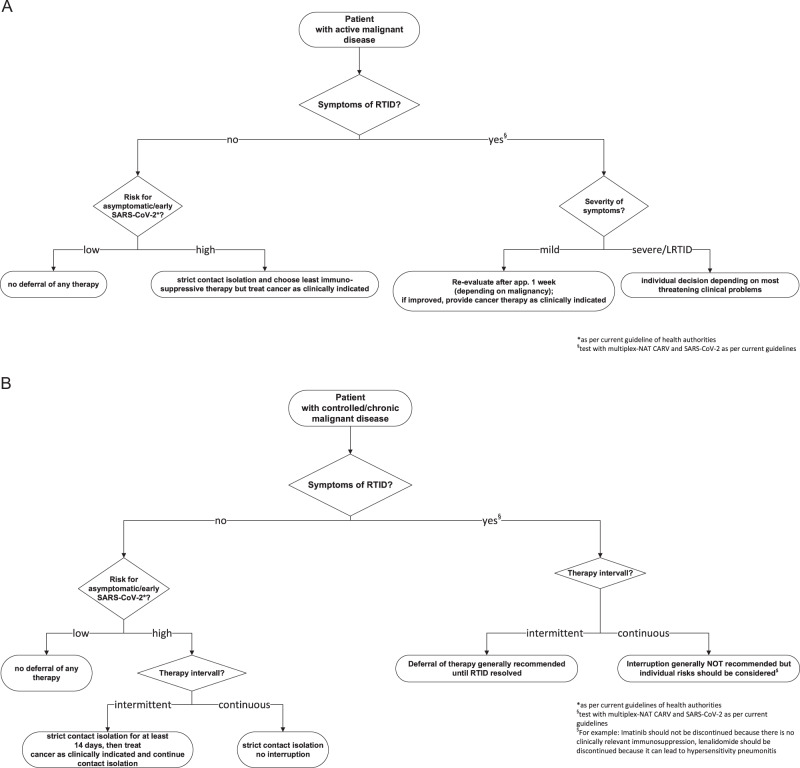
Management of therapy for malignant disease. **a** Proposed algorithm for the management of anti-cancer therapy in patients with active malignant disease. **b** Proposed algorithm for the management of anti-cancer therapy in patients with chronic/controlled malignant disease. RTID respiratory tract infectious disease, LRTID lower respiratory tract infectious disease.

The most relevant and urgent question of clinicians is the most difficult to answer: which anti-cancer therapies should be deferred or interrupted and which should be given or continued? We have attempted to provide a rationale regarding the deferral or interruption of therapies in Fig. 1a, b. Generally, patients with controlled underlying disease have fewer infections than untreated patients [21], therefore, uncontrolled malignant disease should be avoided and life-threatening malignancy needs treatment, especially if there is relevant curative potential. As stated above, impairments and shortages due to the emergency situation need to be taken into account. However, active malignant disease should be treated accordingly and timely to prevent a worsening in outcome. This includes surgical procedures (for example for obstruction) as well as systemic anti-cancer therapy or radiation.

Generally, treatment in asymptomatic patients should continue as close to normal as possible. Prophylactic interruptions of continuous therapies are not generally recommended. Many continuous therapies like for example BCR-ABL targeting TKI are not associated with a clinically relevant increase in immunosuppression and discontinuation may impair control of the malignant disease. Also, clinicians should be aware of the fact that termination of some drugs, for example ruxolitinib, may have devastating effects in terms of a rebound of symptoms [22]. On the other hand, some therapies are associated with significant cytopenias and toxicities which must be assumed as a risk factor for the adverse outcome of COVID-19. In addition, as stated above lymphopenia is likely a specific risk factor for adverse outcome [7]. Therefore, the administration of drugs inhibiting B cells like monoclonal antibodies against CD20 is possibly harmful and should be critically evaluated. Also, drugs associated with hypersensitivity-phenomena may aggravate COVID-19-induced hypersensitivity-pneumonitis [23]. Therefore, interruptions of some therapies may seem prudent in patients presenting with symptoms of RTID. In general, recommendations regarding the management of potential infectious complications of novel and targeted therapies should be observed [24, 25].

**(4) How long should precautions last?**

Precautions in individual cancer patients regarding therapy should last until there are no more clinical signs of ongoing infection and the patient has been tested negative. Clinicians need to be aware of prolonged shedding of CARV in cancer patients [12]. General precautions in the population depend on guidance by national health authorities. Some experts assume that the critical time of the pandemic will last 2–4 months, if measures of clear-cut social distancing are effectively maintained.

**(5) What diagnostic measures should be taken in somebody who shows symptoms of RTID/LRTID?**

In someone presenting with symptoms of RTID, broad diagnostics (ideally SARS-CoV-2 in addition to multiplex-NAT including other CARV like influenza, parainfluenza, metapneumo- and human coronaviruses, and respiratory pathogens like pneumococci) is strongly recommended [11, 26]. Identification of the infectious agent, even if it is not SARS-CoV-2, has therapeutic and regulatory consequences and should thus be obtained. We are aware that national recommendations regarding testing for SARS-CoV-2 may differ and that shortages of tests may become a problem. However, we recommend that cancer patients should be generously tested since it has immediate implications.

Samples should generally be taken from the involved anatomical area. Nasopharyngeal aspirates, samples from lower respiratory tract and nasopharyngeal swabs may be used, nasal swabs alone confer a lower sensitivity [27]. Clinicians should be aware that testing for SARS-CoV-2 may produce false-negative results in asymptomatic or mildly symptomatic patients and in patients with LRTID if samples from the upper respiratory tract are tested [28, 29]. Therefore, it is very important to implement standardized sampling and to repeat tests in patients with unexpected results to avoid bias by pre-analytical mistakes. For diagnosis of LRTID in patients with CARV infection including SARS-CoV-2, CT scans should be used rather than chest X-ray [29–31]. If LRTID is present, patients should undergo standard microbiological testing to test for bacterial or fungal superinfection since superinfection is the most dangerous complication in any CARV-infection [5].

**(6) What general therapeutic measures should be taken in somebody who is infected with SARS-CoV-2?**

It seems logical to reduce immunosuppression in those patients where this is possible. However, possible disadvantages should be taken into consideration like immune reconstitution syndrome [1]. Therefore, in most cases it is probably not prudent to stop immunosuppression. Steroids, particularly high-dose, are generally associated with a prolonged shedding and an increased mortality in CARV-infections [32, 33]. However, recent reports on COVID-19 suggest a benefit of steroid treatment [34] and low-dose steroid (<1 mg/kg/day for 3 days) has been described in an immunosuppressed patient [35]. A randomized trial is currently underway [36]. Thus, it seems reasonable to reduce high doses of steroids to avoid profound immunosuppression by tapering to doses <1 mg/kg/day. As stated above, ivIg-preparations to date are not specifically active against SARS-CoV-2 and are therefore currently not recommended as treatment.

Recent reports support a relevant proportion of hyperinflammation in severe cases of COVID-19 and suggest the consideration of additional anti-inflammatory drugs like tocilizumab or ruxolitinib in patients fulfilling the criteria of HLH [1, 37–39]. Also, because of the increased incidence of pulmonary embolism, anticoagulation is strongly recommended [40].

**(7) How can COVID 19 be treated specifically?**

There are no established standard treatments for COVID-19. Whenever possible, patients should be included into clinical trials. Infectious disease specialists should be involved in therapeutic decisions. If experimental treatment with drugs approved for other indications is considered, physicians should share the decision with the patients and inform them about the chances and risks of such strategies.

Experiences with SARS and first experiences with SARS-CoV-2 suggest the efficacy of some treatment options [41]: lopinavir/ritonavir, chloroquine, and remdesivir seem most promising. Ribavirin is potentially effective based on *in silico* models, but no clinical data is available. For SARS, retrospective data with low quality of evidence suggested the effectiveness of ribavirin in combination with lopinavir/ritonavir.

If specific treatment is administered it should probably be given as early as possible to be effective, similar to treatment of influenza.

**(8) What else should be considered?**

Since cancer patients are particularly prone to severe courses of CARV-infections, clinicians should be aware of other CARV and other respiratory pathogens, which may require specific treatment [11, 26, 42]. Also, co-infections are much more common in cancer patients and as stated above, cancer patients tend to take considerably longer to clear the virus than healthy controls [12]. Often the question is raised whether blood products may confer a risk of infection—this does not seem to be the case. Therefore, blood products are safe, although they might become rare because of restrictions of donors.

## Special aspects in pediatric hematology/oncology

**(9) What are the symptoms in children and how similar are they to those in adults?**

Clinical manifestation in healthy children has been reported to be similar to adults, with cough and fever the most frequent symptoms. Presentation is usually milder than in adults. Rarely, also gastrointestinal symptoms such as diarrhea, nausea, vomiting, and feeding difficulties may be present [43]. In immunocompromised patients, some possible differences may be noted due to lymphopenia and pre-existing conditions (pulmonary cGvHD) (same as in the adults) resulting in a more severe presentation and rapid deterioration of the general condition.

**(10) What is the risk of a child getting infected?**

As shown by data from China, COVID-19 seems to be uncommon in children (0.9–1.2%) compared to adults [44].

**(11) When should a child be tested and how?**

Due to the clinical evidence of frequent mild onset and evolution, only children with moderate-severe clinical condition admitted to the hospital for care are currently tested for COVID-19 infection (Italian experience, specific indications could be different according to National Authorities). Due to the atypical presentation and worse outcome in immunocompromised children, testing could be advised even with mild symptoms (local strategy) because of possible therapeutic implications. Diagnostic principles are the same as in adults including additional work-up in all patients with LRTID.

**(12) Should we screen all children before chemotherapy?**

Because of the indeterminate onset in children, some centers may decide to screen all children prior to immunosuppressive therapy. Another option could be to screen children only if they are at high risk as defined by national health authorities (for example recent contact with COVID-19 positive person or if family members are symptomatic (cough, fever, running nose, or diarrhea)).

**(13) Special characteristics of the pediatric infection**

There seems to be a persistence of positive rectal swab even in asymptomatic children, which can lead to oral–fecal transmission [45, 46]. Also, results from imaging procedures may be moderately different [43, 47]. This should be taken into account when local policies for management of COVID-19 in the respective departments are devised.

## Summary

COVID-19, caused by SARS-CoV-2, is expected to be a devastating infection in patients with active cancer. It should be taken seriously and managed rigorously without jeopardizing the curative chance of individual cancer patients. In view of the rapidly changing evidence and general situation, we attempted to provide current and clinically relevant guidance on the management of cancer patients with or at risk of COVID-19.

## References

[1] Mehta P, McAuley DF, Brown M, Sanchez E, Tattersall RS, Manson JJ. COVID-19: consider cytokine storm syndromes and immunosuppression. Lancet. 2020;395:1033–4. 10.1016/S0140-6736(20)30628-0 PMC7270045 32192578

[2] La Rosee P, Horne A, Hines M, von Bahr Greenwood T, Machowicz R, Berliner N (2019). Recommendations for the management of hemophagocytic lymphohistiocytosis in adults. Blood.

[3] Chen J, Wang X, Zhang S. Findings of acute pulmonary embolism in COVID-19 patients. Lancet Infect Dis. 2020. https://ssrn.com/abstract=3548771.

[4] Ison MG, Hirsch HH. Community-acquired respiratory viruses in transplant patients: diversity, impact, unmet clinical needs. Clin Microbiol Rev. 2019. 10.1128/CMR.00042-19. 10.1128/CMR.00042-19 PMC7399564 31511250

[5] Hermann B, Lehners N, Brodhun M, Boden K, Hochhaus A, Kochanek M (2017). Influenza virus infections in patients with malignancies–characteristics and outcome of the season 2014/15. A survey conducted by the Infectious Diseases Working Party (AGIHO) of the German Society of Haematology and Medical Oncology (DGHO). Eur J Clin Microbiol Infect Dis.

[6] Guan WJ, Ni ZY, Hu Y, Liang WH, Ou CQ, He JX, et al. Clinical characteristics of coronavirus disease 2019 in China. N Engl J Med. 2020. 10.1056/NEJMoa2002032. 10.1056/NEJMoa2002032 PMC7092819 32109013

[7] Zhou F, Yu T, Du R, Fan G, Liu Y, Liu Z, et al. Clinical course and risk factors for mortality of adult inpatients with COVID-19 in Wuhan, China: a retrospective cohort study. Lancet. 2020;395:1054–62. 10.1016/S0140-6736(20)30566-3 PMC7270627 32171076

[8] Liang W, Guan W, Chen R, Wang W, Li J, Xu K (2020). Cancer patients in SARS-CoV-2 infection: a nationwide analysis in China. Lancet Oncol.

[9] Xia Y, Jin R, Zhao J, Li W, Shen H. Risk of COVID-19 for cancer patients. Lancet Oncol. 2020;21:PE181. 10.1016/S1470-2045(20)30150-9 PMC7130057 32142622

[10] Battegay M, Kuehl R, Tschudin-Sutter S, Hirsch HH, Widmer AF, Neher RA (2020). 2019-novel Coronavirus (2019-nCoV): estimating the case fatality rate - a word of caution. Swiss Med Wkly.

[11] Hirsch HH, Martino R, Ward KN, Boeckh M, Einsele H, Ljungman P (2013). Fourth European Conference on Infections in Leukaemia (ECIL-4): guidelines for diagnosis and treatment of human respiratory syncytial virus, parainfluenza virus, metapneumovirus, rhinovirus, and coronavirus. Clin Infect Dis.

[12] Lehners N, Tabatabai J, Prifert C, Wedde M, Puthenparambil J, Weissbrich B (2016). Long-term shedding of influenza virus, parainfluenza virus, respiratory syncytial virus and nosocomial epidemiology in patients with hematological disorders. PLoS ONE.

[13] Jefferson T, Del Mar CB, Dooley L, Ferroni E, Al-Ansary LA, Bawazeer GA, et al. Physical interventions to interrupt or reduce the spread of respiratory viruses. Cochrane Database Syst Rev. 2011;7:CD006207. 10.1002/14651858.CD006207.pub4 PMC6993921 21735402

[14] EMA. Guideline on core SmPC for human normal immunoglobulin for intravenous administration (IVIg). 2018.

[15] Ogimi C, Krantz EM, Golob JL, Waghmare A, Liu C, Leisenring WM (2018). Antibiotic exposure prior to respiratory viral infection is associated with progression to lower respiratory tract disease in allogeneic hematopoietic cell transplant recipients. Biol Blood Marrow Transplant.

[16] Mikulska M, Averbuch D, Tissot F, Cordonnier C, Akova M, Calandra T (2018). Fluoroquinolone prophylaxis in haematological cancer patients with neutropenia: ECIL critical appraisal of previous guidelines. J Infect.

[17] Neumann S, Krause SW, Maschmeyer G, Schiel X, von Lilienfeld-Toal M (2013). Primary prophylaxis of bacterial infections and Pneumocystis jirovecii pneumonia in patients with hematological malignancies and solid tumors: guidelines of the Infectious Diseases Working Party (AGIHO) of the German Society of Hematology and Oncology (DGHO). Ann Hematol.

[18] Vehreschild JJ, Bohme A, Cornely OA, Kahl C, Karthaus M, Kreuzer KA (2014). Prophylaxis of infectious complications with colony-stimulating factors in adult cancer patients undergoing chemotherapy-evidence-based guidelines from the Infectious Diseases Working Party AGIHO of the German Society for Haematology and Medical Oncology (DGHO). Ann Oncol.

[19] Cordonnier C, Einarsdottir S, Cesaro S, Di Blasi R, Mikulska M, Rieger C (2019). Vaccination of haemopoietic stem cell transplant recipients: guidelines of the 2017 European Conference on Infections in Leukaemia (ECIL 7). Lancet Infect Dis.

[20] Mikulska M, Cesaro S, de Lavallade H, Di Blasi R, Einarsdottir S, Gallo G (2019). Vaccination of patients with haematological malignancies who did not have transplantations: guidelines from the 2017 European Conference on Infections in Leukaemia (ECIL 7). Lancet Infect Dis.

[21] Brioli A, Klaus M, Sayer H, Scholl S, Ernst T, Hilgendorf I (2019). The risk of infections in multiple myeloma before and after the advent of novel agents: a 12-year survey. Ann Hematol.

[22] Tefferi A, Pardanani A (2011). Serious adverse events during ruxolitinib treatment discontinuation in patients with myelofibrosis. Mayo Clin Proc.

[23] Song YG, Shin HS. COVID-19, a clinical syndrome manifesting as hypersensitivity pneumonitis. Infect Chemother. 2020;52:110–2. 10.3947/ic.2020.52.1.110 PMC7113449 32153144

[24] Maschmeyer G, De Greef J, Mellinghoff SC, Nosari A, Thiebaut-Bertrand A, Bergeron A (2019). Infections associated with immunotherapeutic and molecular targeted agents in hematology and oncology. A position paper by the European Conference on Infections in Leukemia (ECIL). Leukemia.

[25] Steegmann JL, Baccarani M, Breccia M, Casado LF, Garcia-Gutierrez V, Hochhaus A (2016). European LeukemiaNet recommendations for the management and avoidance of adverse events of treatment in chronic myeloid leukaemia. Leukemia.

[26] von Lilienfeld-Toal M, Berger A, Christopeit M, Hentrich M, Heussel CP, Kalkreuth J (2016). Community acquired respiratory virus infections in cancer patients-Guideline on diagnosis and management by the Infectious Diseases Working Party of the German Society for haematology and Medical Oncology. Eur J Cancer.

[27] Wang W, Xu Y, Gao R, Lu R, Han K, Wu G, et al. Detection of SARS-CoV-2 in different types of clinical specimens. JAMA. 2020. 10.1001/jama.2020.3786. 10.1001/jama.2020.3786 PMC7066521 32159775

[28] Xie X, Zhong Z, Zhao W, Zheng C, Wang F, Liu J. Chest CT for typical 2019-nCoV pneumonia: relationship to negative RT-PCR testing. Radiology. 2020. 10.1148/radiol.2020200343. 10.1148/radiol.2020200343 PMC7233363 32049601

[29] Ai T, Yang Z, Hou H, Zhan C, Chen C, Lv W, et al. Correlation of chest CT and RT-PCR testing in coronavirus disease 2019 (COVID-19) in China: a report of 1014 cases. Radiology. 2020. 10.1148/radiol.2020200642. 10.1148/radiol.2020200642 PMC7233399 32101510

[30] Mayer JL, Lehners N, Egerer G, Kauczor HU, Heussel CP (2014). CT-morphological characterization of respiratory syncytial virus (RSV) pneumonia in immune-compromised adults. RoFo.

[31] Li Y, Xia L. Coronavirus disease 2019 (COVID-19): role of chest CT in diagnosis and management. AJR Am J Roentgenol. 2020. 10.2214/AJR.20.22954. 10.2214/AJR.20.22954 32130038

[32] Lansbury LE, Rodrigo C, Leonardi-Bee J, Nguyen-Van-Tam J, Lim WS. Corticosteroids as adjunctive therapy in the treatment of influenza: an updated Cochrane systematic review and meta-analysis. Crit Care Med. 2019;3:CD010406. 10.1097/CCM.0000000000004093 31939808

[33] Stockman LJ, Bellamy R, Garner P (2006). SARS: systematic review of treatment effects. PLoS Med..

[34] Wu C, Chen X, Cai Y, Xia J, Zhou X, Xu S, et al. Risk factors associated with acute respiratory distress syndrome and death in patients with coronavirus disease 2019 pneumonia in Wuhan, China. JAMA Intern Med. 2020. 10.1001/jamainternmed.2020.0994. 10.1001/jamainternmed.2020.0994 PMC7070509 32167524

[35] Zhu F, Cao Y, Xu S, Zhou M. Co-infection of SARS-CoV-2 and HIV in a patient in Wuhan City, China. J Med Virol. 2020. 10.1002/jmv.25732. 10.1002/jmv.25732 PMC7228399 32160316

[36] Zhou YH, Qin YY, Lu YQ, Sun F, Yang S, Harypursat V, et al. Effectiveness of glucocorticoid therapy in patients with severe novel coronavirus pneumonia: protocol of a randomized controlled trial. Chin Med J. 2020. 10.1097/CM9.0000000000000791.

[37] Birndt S, Schenk T, Heinevetter B, Brunkhorst FM, Maschmeyer G, Rothmann F (2020). Hemophagocytic lymphohistiocytosis in adults: collaborative analysis of 137 cases of a nationwide German registry. J Cancer Res Clin Oncol.

[38] La Rosee P (2016). Alleviating the storm: ruxolitinib in HLH. Blood.

[39] Stebbing J, Phelan A, Griffin I, Tucker C, Oechsle O, Smith D, et al. COVID-19: combining antiviral and anti-inflammatory treatments. Lancet Infect Dis. 2020;20:400–2. 10.1016/S1473-3099(20)30132-8 PMC7158903 32113509

[40] Zhai Z, Li C, Chen Y, Gerotziafas G, Zhang Z, Wan J, et al. Prevention Treatment of VTE Associated with COVID-19 Infection Consensus Statement Group, Pulmonary Embolism Pulmonary Vascular Diseases Group of the Chinese Thoracic Society, Pulmonary Embolism Pulmonary Vascular Disease Working Committee of Chinese Association of Chest Physicians, National Cooperation Group on Prevention Treatment of Pulmonary Embolism Pulmonary Vascular Disease, National Program Office for Prevention Treatment of Pulmonary Embolism Deep Vein Thrombosis, China Grade Center, Evidence-based Medicine Center of School of Basic Medical Sciences of Lanzhou University. Prevention and Treatment of Venous Thromboembolism Associated with Coronavirus Disease 2019 Infection: A Consensus Statement before Guidelines. Thromb Haemost. 2020. 10.1055/s-0040-1710019. [Epub ahead of print]

[41] Martinez MA. Compounds with therapeutic potential against novel respiratory 2019 coronavirus. Antimicrob Agents Chemother. 2020. 10.1128/AAC.00399-20. 10.1128/AAC.00399-20 PMC7179632 32152082

[42] Engelhard D, Mohty B, de la Camara R, Cordonnier C, Ljungman P (2013). European guidelines for prevention and management of influenza in hematopoietic stem cell transplantation and leukemia patients: summary of ECIL-4 (2011), on behalf of ECIL, a joint venture of EBMT, EORTC, ICHS, and ELN. Transpl Infect Dis.

[43] Xia W, Shao J, Guo Y, Peng X, Li Z, Hu D. Clinical and CT features in pediatric patients with COVID-19 infection: different points from adults. Pediatr Pulmonol. 2020;55:1169–74. 10.1002/ppul.24718 PMC7168071 32134205

[44] Lee PI, Hu YL, Chen PY, Huang YC, Hsueh PR. Are children less susceptible to COVID-19? J Microbiol Immunol Infect. 2020. pii: S1684-1182(20)30039-6. 10.1016/j.jmii.2020.02.011. [Epub ahead of print] 10.1016/j.jmii.2020.02.011 PMC7102573 32147409

[45] Zhang W, Du RH, Li B, Zheng XS, Yang XL, Hu B (2020). Molecular and serological investigation of 2019-nCoV infected patients: implication of multiple shedding routes. Emerg Microbes Infect.

[46] Xu Y, Li X, Zhu B, Liang H, Fang C, Gong Y, et al. Characteristics of pediatric SARS-CoV-2 infection and potential evidence for persistent fecal viral shedding. Nat Med. 2020;20:502–5. 10.1038/s41591-020-0817-4 PMC7095102 32284613

[47] Li W, Cui H, Li K, Fang Y, Li S. Chest computed tomography in children with COVID-19 respiratory infection. Pediatr Radiol. 2020. 10.1007/s00247-020-04656-7. 10.1007/s00247-020-04656-7 PMC7080075 32162081

